# Association between paraoxonase 1 -108C/T polymorphism and coronary heart disease: an updated meta-analysis

**DOI:** 10.3389/fcvm.2024.1339701

**Published:** 2024-08-01

**Authors:** Jiadan Liao, Pengcheng Wang

**Affiliations:** ^1^ Department of Cardiology, The Third Affiliated Hospital of Zhejiang Chinese Medical University, Hangzhou, China; ^2^ Department of Tuberculosis, Hangzhou Red Cross Hospital, Hangzhou, China

**Keywords:** coronary heart disease, -108C/T, risk, meta-analysis, PON1

## Abstract

**Background:**

At present, no consensus is reached among articles that investigate the relationship of paraoxonase 1(PON1) -108C/T polymorphism with susceptibility of coronary heart disease (CHD) so far. In this regard, the present meta-analysis was conducted to comprehensively review existing articles related to the relationship of PON1 -108C/T polymorphism with CHD susceptibility. It was preregistered in the International Platform of Registered Systematic Review and Meta-Analysis Protocols (INPLASY)-INPLASY202430117.

**Methods:**

Articles that explored the relationship between PON1 -108C/T polymorphism and CHD incidence were searched from electronic databases according to our preset study selection criteria. Thereafter, we adopted stata 12.0 software to analyze our screened studies. At the same time, odds ratios (ORs) and related 95% confidence intervals (95% CIs) were determined for evaluating association strength.

**Results:**

At last, this meta-analysis selected altogether 13 case-control studies that involved 2,979 cases and 2,887 control subjects. We found that the *PON1* -108C/T polymorphism displayed marked relationship with CHD susceptibility (T vs. C: OR = 1.24, 95% CI 1.07–1.45; CT vs. CC: OR = 1.33, 95% CI 1.17–1.52; TT vs. CC: OR = 1.51, 95% CI 1.09–2.09; Recessive model: OR = 1.16, 95% CI 0.93–1.45; Dominant model: OR = 1.45, 95% CI 1.16–1.81). Moreover, subgroup analysis showed that race and sample size had no impact on the results. Bioinformatics analysis showed that -108C>T polymorphism was relation to PON1 gene expression (https://gtexportal.org/home/).

**Conclusions:**

The *PON1* -108T allele is identified as the possible low-penetrant risk factor of CHD, as suggested by our present meta-analysis.

**Systematic Review Registration:** https://inplasy.com/inplasy-2024-3-0117/, Identifier INPLASY202430117.

## 1 Introduction

Coronary heart disease (CHD) represents a leading cause resulting in disability and mortality worldwide, which also becomes a main public health issue (1). Although our knowledge of CHD has advanced considerably, the detailed pathogenesis of CHD remains unclear. A series of modifiable and non-modifiable risk factors can influence the formation of atherosclerotic plaque, including age, gender, smoking habit, intake of alcohol, physical inactivity, overweight/obesity, abnormal lipid profile, high blood pressure, as well as family history of CHD (2, 3). Paraoxonase (PON) family includes PON1, PON2, and PON3. PONl and PON3 belong to secretory enzymes, which are mainly expressed in liver and kidney tissues, and are closely bound to high-density lipoprotein in serum. PON2 belongs to intracellular enzymes and can be widely expressed in various tissue cells. In PON family, domestic and foreign scholars have conducted the most comprehensive and in-depth research on PON1. PON1 is the HDL-related Ca2+ dependent glycoprotein (lactonase), which presents antioxidant and anti-atherogenic features through paraoxon hydrolysis (4).

PON1 is an HDL-associated esterase that hydrolyses lipoperoxides. It acts as a protective factor against oxidative modifications of LDL, indicating that it possibly exerts an important part in preventing atherosclerotic process. This has led to the hypothesis that the lower the PON1 activity is, the higher will be the accumulation of oxidized LDL and risk of CHD. Recent meta-analysis showed that PON-1 activity is significantly lower in CAD patients. In human being, PON1 locates within q21.3–q22.1 in chromosome 7 long arm. The PON1 gene has nearly 200 single nucleotide polymorphisms (SNPs). Notably, the *PON1* -108 polymorphism promoter region [c.-108C>T (C: cytosine T: thymine); dbSNP: rs705379; OMIM database: +1688200003] greatly affects PON1 function. Besides, in certain cellular systems, the -108C/T polymorphism of this gene takes up 23% expression of PON1, while -108TT constructs decreased PON1 level relative to -108CC constructs.

Many articles are conducted to analyze the association of *PON1* -108C>T polymorphisms with CHD susceptibility, however, no consensus results are obtained so far. According to a previous systematic review, the *PON1* -108C>T may be potential CHD risk factors. Due to three missing eligible studies, this meta-analysis did not include all potentially relevant studies. In addition, previous meta-analysis did not use subgroup analysis and bioinformatics analysis. To evaluate the impact of *PON1* -108C>T polymorphism on CHD clinical outcomes, we conducted the current updated meta-analysis.

## 2 Methods

### 2.1 Search strategy

This meta-analysis was implemented according to PRISMA checklist (Attachment 1). With the guidance of Preferred Reporting Items for Systematic Review and Meta-Analysis Protocols guidelines we has completed our meta-analysis protocol, and has been registered in the International Platform of Registered Systematic Review and Meta-analysis Protocols (INPLASY) with the number is INPLASY202430117. Web of Science, PubMed, CNKI and Embase were systemically retrieved to enroll eligible articles published before June 2020. The keywords shown below were used, including “paraoxonase 1”, “paraoxonase-1”, “PON 1”, “PON-1”, “variant”, “variation”, “mutation”, “SNP”, “polymorphism”, “atherosclerosis”, “arteriosclerosis”, “coronary heart disease”, “coronary artery disease”, “acute coronary syndrome”, “myocardialinfarction”. Besides, reference lists were manually searched to prevent omitting any eligible study.

### 2.2 Inclusion and exclusion

We selected studies based on the following criteria: (a) case-control articles evaluating the relationship of *PON1* -108C>T with CHD susceptibility; (b) studies conducted by enrolling irrelevant subjects; (c) studies with enough data on ORs and 95% CIs. At the same time, reports, reviews, letters and comments were eliminated from this study. For duplicates, the one that had the greatest sample size was adopted for the present meta-analysis.

### 2.3 Data extraction

Two independent investigators collected data and were also responsible for result checking. Data extracted were as follows, first author, publication year, area, case/control numbers, frequencies of genotypes among cases/controls, along with Hardy-Weinberg equilibrium (HWE) evidence among controls. Any disagreement between them was settled down by mutual negotiation.

### 2.4 Statistical analysis

HWE among controls of every article was assessed through chi-square test, with *P* < 0.05 indicating significance of disequilibrium. Besides, ORs and 95%CIs were adopted for estimating the association strength of *PON1* -108C>T polymorphism with CHD risk through allele model (T vs. C), heterozygote comparison (CT vs. CC), homozygote comparison (TT vs. CC), recessive model (TT vs. CT + CC) and dominant model (TT + CT vs. CC). In addition, chi-square test was conducted to quantify the heterogeneity influence, with I^2^ > 50% suggesting that there was heterogeneity among different studies, as a result, a random effects model should be selected; else, the fixed effects model should be used. We also conducted sensitivity for evaluating result stability through eliminating a study each time for determining its impact on combined ORs. Furthermore, Begg's funnel plot was employed to investigate the bias of publication, with *P *< 0.05 indicating the presence of significant bias of publication. STATA12.0 (Stata Corporation, College Station, Texas) was utilized for meta-analysis.

### 2.5 In-silico analysis

Using the GTEx expression quantitative trait locus (eQTL) data, -108C>T polymorphism was examined in relation to *PON1* gene expression (https://gtexportal.org/home/).

## 3 Results

### 3.1 Characteristics of included studies

**Figure 1:**
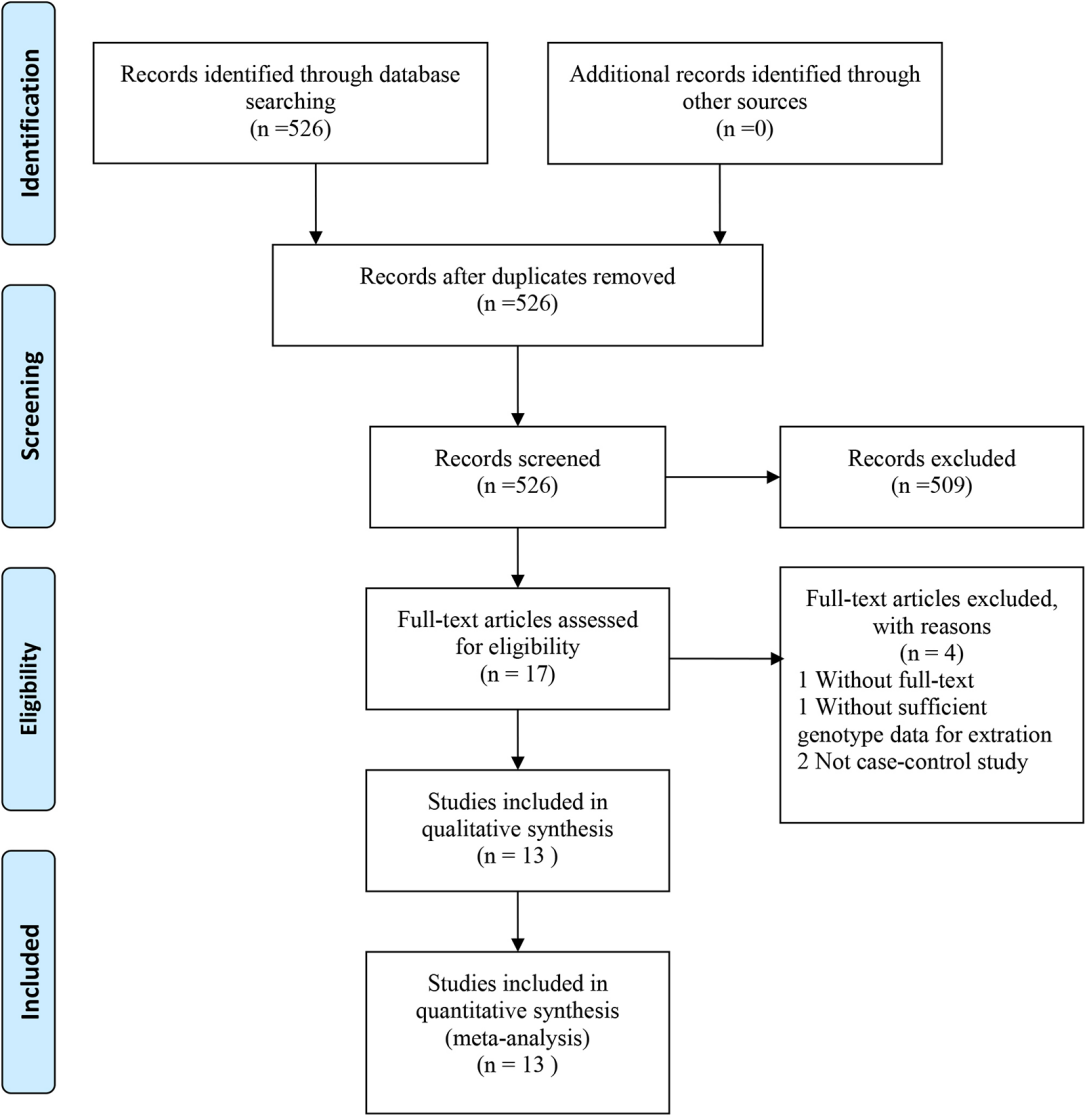
The flowchart of the included studies in the meta-analysis.

Figure 1 presents the study screening procedure. Altogether 526 relevant papers were identified from Web of Science, PubMed, CNKI and Embase. Finally, we enrolled 13 case-control studies into the present meta-analysis (5–14). These studies were published between 2000 and 2020. The detailed information of the included papers was demonstrated in Table 1. Of these, four studies focused on Caucasians, nine studies focused on Asians. The genetic distributions of controls conformed to HWE among the enrolled studies, with the only exception of Ahmad et al. The general features of the eight researches were summarized in Table 2.

**Table 1:** Summary ORs and 95% CI of PON1 -108C>T polymorphism with CHD risk.

Variables	*N* ^a^	T vs. C	TT vs. CC	CT vs. CC	Dominant model	Recessive model
		OR (95% CI) Model	OR (95% CI) Model	OR (95% CI) Model	OR (95% CI) Model	OR (95% CI) Model
Total	13	1.24 (1.07–1.45) R	1.51 (1.09–2.09) R	1.33 (1.17–1.52) F	1.45 (1.16–1.81) R	1.16 (0.93–1.45) R
Ethnicity						
Asian	9	1.29 (1.03–1.61) R	1.61 (0.99–2.60) R	1.52 (1.16–1.99) R	1.58 (1.13–2.21) R	1.14 (0.83–1.56) R
Caucasian	4	1.16 (1.02–1.32) F	1.37 (0.96–1.97) R	1.25 (1.01–1.55) F	1.29 (1.05–1.57) F	1.19 (0.88–1.60) R
Sample size						
>500	5	1.32 (1.04–1.66) R	1.62 (0.96–2.72) R	1.39 (1.07–1.82) R	1.48 (1.04–2.12) R	1.28 (0.90–1.83) R
≤500	8	1.19 (0.96–1.46) R	1.51 (1.09–2.09) R	1.33 (1.09–1.63) F	1.30 (1.07–1.58) F	1.06 (0.80–1.41) R
HWE						
Yes	12	1.29 (1.11–1.50) R	1.63 (1.18–2.25) R	1.33 (1.17–1.52) F	1.45 (1.16–1.81) R	1.16 (0.93–1.45) R
No	1	–	–	–	–	–

^a^ Number of comparisons.

**Table 2:** Included studies of the PON1 -108C>T polymorphism with CHD.

First author/year	Country	Ethnicity	*n*	Case		Control		Case			Control			HWE test
				C	T	C	T	CC	CT	TT	CC	CT	TT	
James 2000	France	Caucasian	137/273	120	154	278	268	25	70	42	63	152	58	0.06
Huo 2002	China	Asian	60/150	46	74	144	156	7	32	21	39	66	45	0.14
Wang 2003	China	Asian	474/475	404	544	420	530	160	222	92	155	219	101	0.15
Blatter 2006	Switzerland	Caucasian	706/199	678	734	188	210	164	354	188	48	90	61	0.20
Saeed 2007	Pakistan	Caucasian	203/357	186	220	378	336	40	101	62	106	165	86	0.17
Ahmad 2011	India	Asian	204/178	235	173	185	171	67	101	36	54	77	47	0.01
Gupta 2011	India	Asian	350/300	302	398	342	258	68	166	116	100	140	60	0.39
Gupta 2012	India	Asian	300/250	233	367	258	242	48	137	115	73	112	65	0.10
Shao 2006	China	Asian	93/128	65	121	122	134	9	47	30	32	58	38	0.30
Shao 2007	China	Asian	52/128	38	66	122	134	5	28	19	32	58	38	0.30
Strauss 2005	Poland	Caucasian	174/117	177	171	126	108	90	174	84	68	116	50	0.97
Li 2009	China	Asian	206/292	210	202	278	306	45	120	41	66	146	80	0.97
Chen 2012	China	Asian	20/40	20	20	44	36	5	10	5	12	20	8	0.95

HWE, Hardy-Weinberg equilibrium.

### 3.2 Meta-analysis results

**Figure 2:**
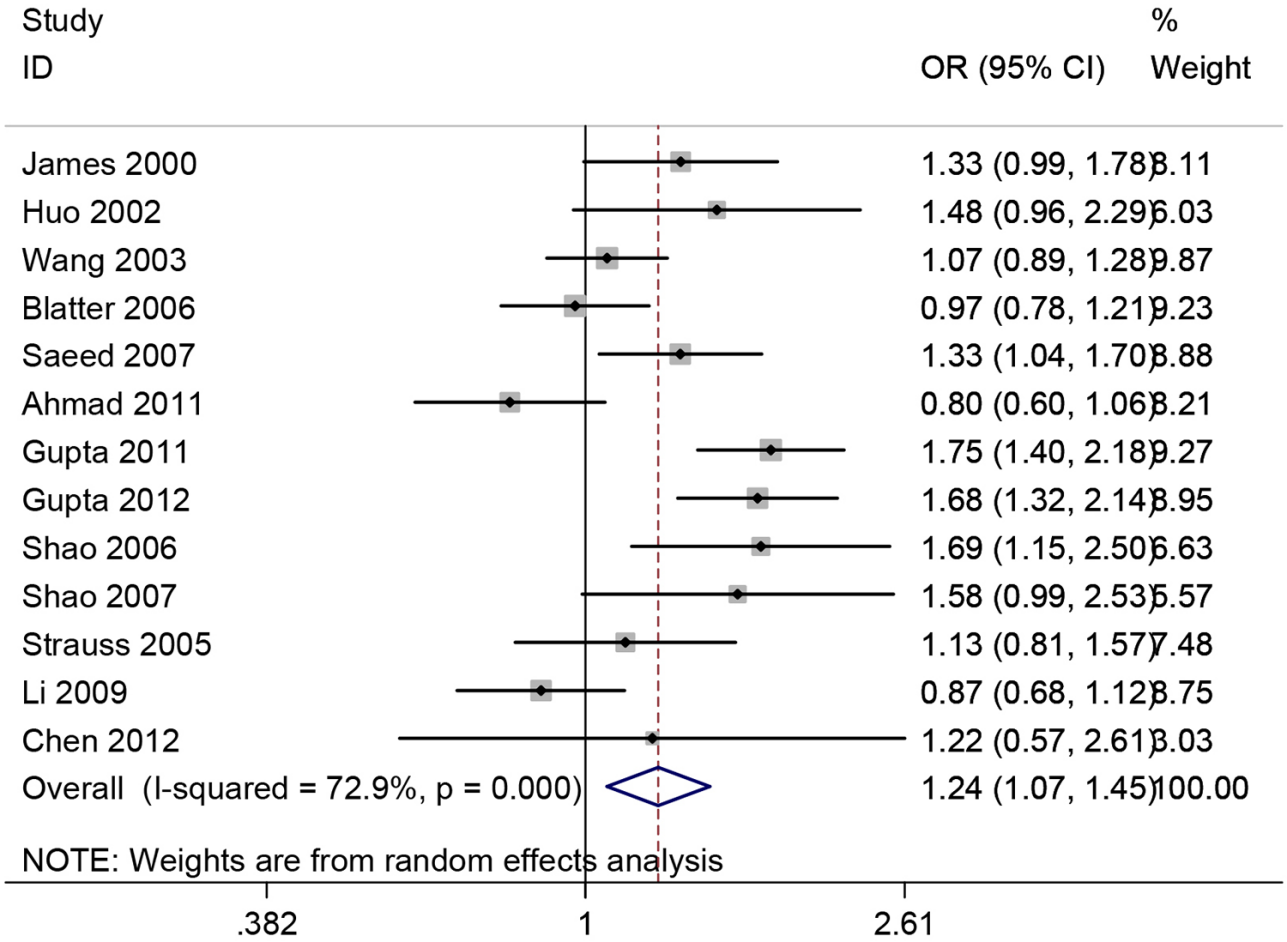
Forest plot for meta-analysis of the association between the PON1 -108C/T and CHD risk.

**Figure 3:**
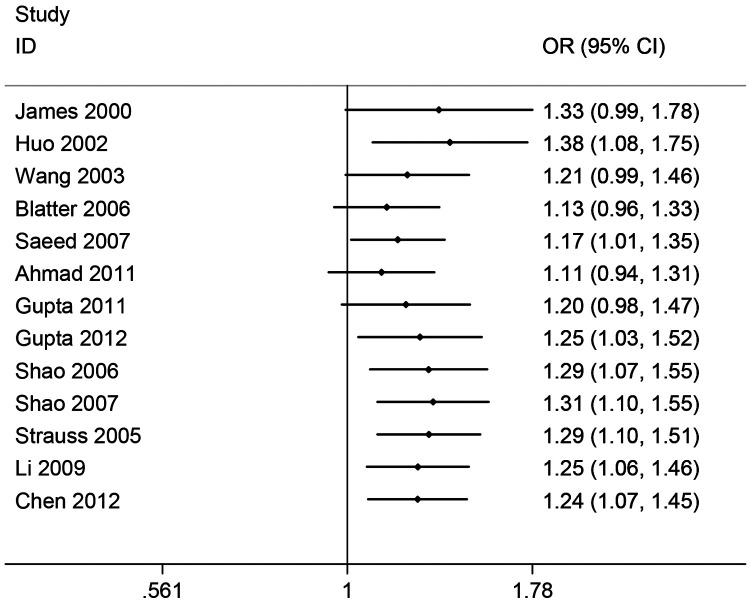
Cumulative meta-analysis for PON1 -108C/T polymorphism and CHD.

Table 1 and Figure 2 display the association of *PON1* -108C>T polymorphism with CHD incidence. It was observed that, *PON1* -108T polymorphism showed significant correlation with CHD incidence (T vs. C: OR = 1.24, 95% CI 1.07–1.45; CT vs. CC: OR = 1.33, 95% CI 1.17–1.52; TT vs. CC: OR = 1.51, 95% CI 1.09–2.09; Recessive model: OR = 1.16, 95% CI 0.93–1.45; Dominant model: OR = 1.45, 95% CI 1.16–1.81). In addition, significant association was found from subgroup analyses based on HWE, sample size and race. Sensitivity analysis was also conducted, which suggested that none of the enrolled studies had significant influence on the combined ORs, indicating our result stability. The cumulative meta-analyses showed a increasing trend in the estimated risk effect, which showed *PON1* -108 polymorphism is associated with HCC risk, and the results were stable (Figure 3).

### 3.3 Sensitivity analysis

**Figure 4:**
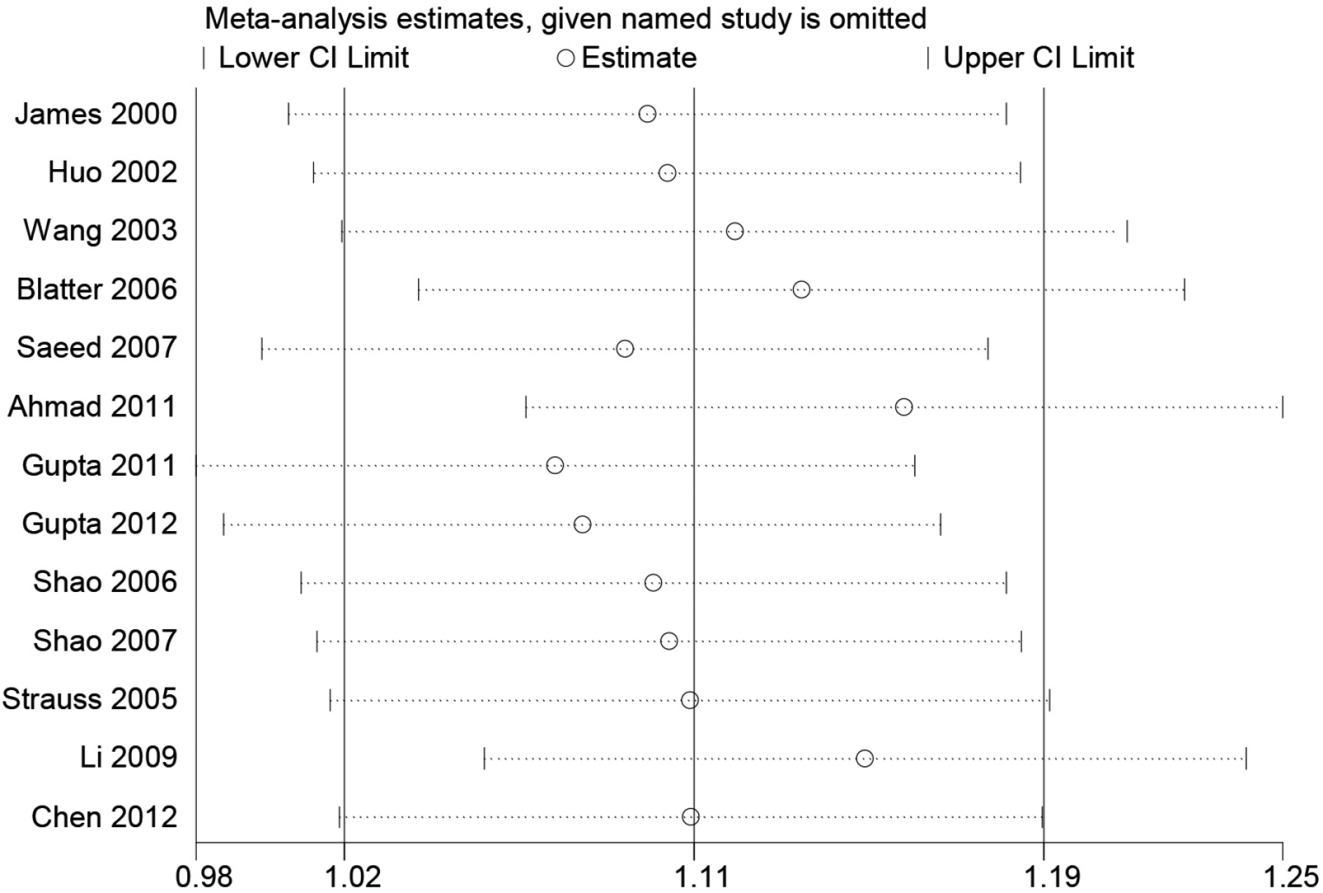
Sensitivity analysis of the association between the PON1 -108C/T and CHD risk.

Sensitivity analysis was performed by omitting each study in turn to evaluate the influence of single studies on the overall estimation (Figure 4). The corresponding pooled OR and significant results did not change materially, indicating that our results were statistically robust.

### 3.4 Publication bias

**Figure 5:**
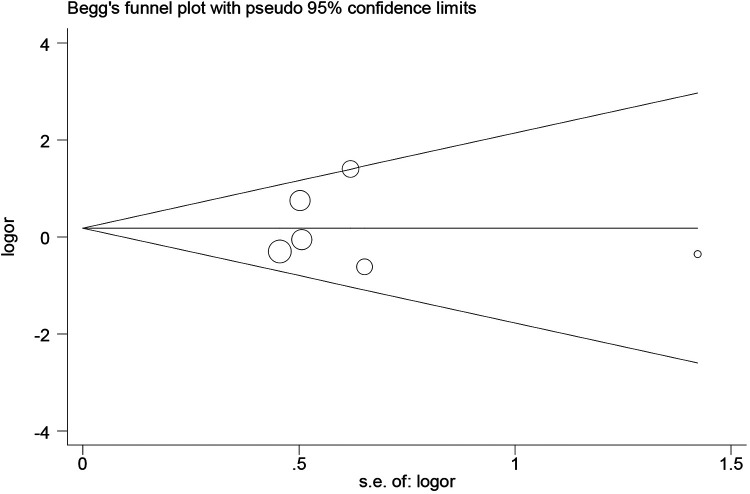
Begg's funnel plot analysis of the PON1 -108C/T polymorphism.

We visualized the funnel plot to analyze the potential bias of publication, and no obvious bias of publication was detected (Figure 5), which indicated that the present meta-analysis had low bias of publication.

### 3.5 Functional predictions

**Figure 6:**
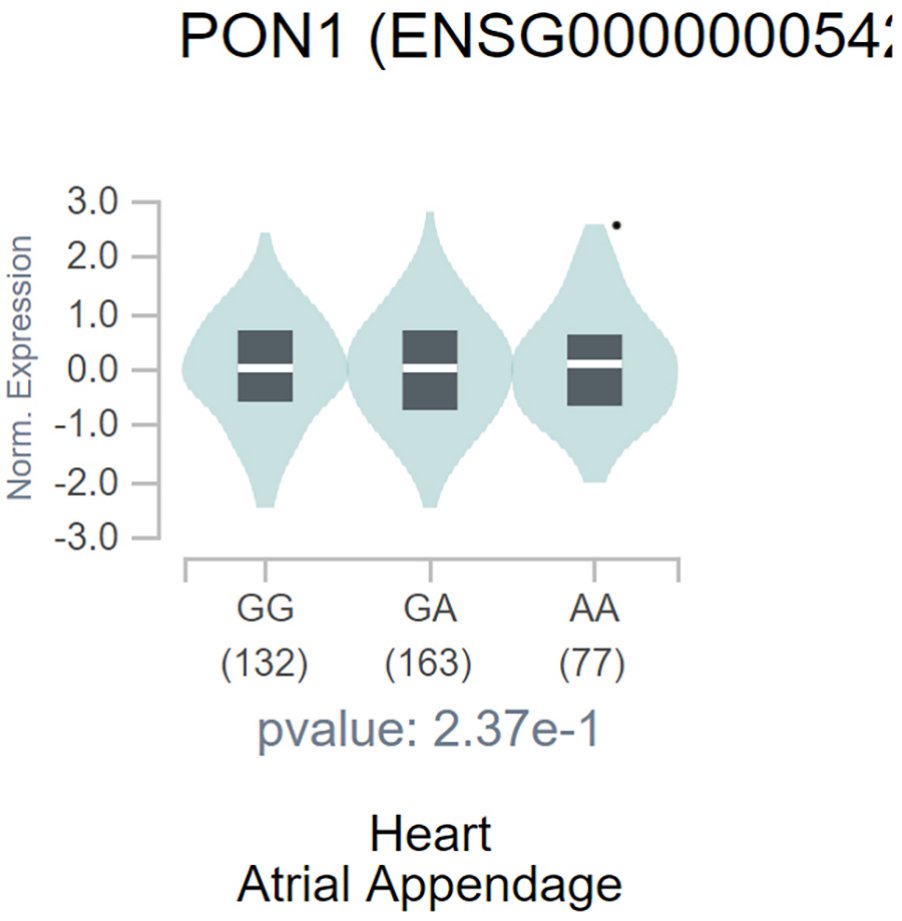
Violin plot shows the correlation between PON1 -108C/T polymorphism with PON1 expression. The figure was from the GTEx.

The GTEx portal shows a significant correlation between *PON1* -108C>T polymorphism and heart PON1 gene expression. In detail, the T allele of *PON1* -108C>T polymorphism significantly correlated with decreased PON1 expression (Figure 6).

## 4 Discussion

CHD represents a disorder that involves multiple factors, and its etiology remains largely unclear at present. It has been increasingly suggested that CHD results from the complicated interactions among genetic and environmental factors. PON1, an enzyme related to HDL-C, can resist the peroxidation of LDL-C. The decreased PON1 activity has been suggested to induce an increased incidence of coronary atherosclerosis (12). Besides, -108TT has been previously reported to down-regulate the PON1 level relative to the -108CC constructs (7). In this regard, we predicted that *PON1* -108T might participate in CHD incidence. *PON1* -108C>T polymorphism is previously suggested to be related to CHD incidence, yet no consistent findings are obtained. For clarifying those heterogeneous results, the present meta-analysis was conducted aiming to examine the association between *PON1* -108C>T polymorphism and CHD susceptibility.

This meta-analysis enrolled 13 case-control studies that involved 2,979 patients along with 2,887 controls, which aimed to examine the association between *PON1* -108C>T polymorphism and CHD susceptibility. As far as we know, the present meta-analysis was the first to comprehensively assess the correlation between *PON1* -108C>T polymorphism and CHD susceptibility. Overall, *PON1* -108C>T polymorphism was significantly associated with CHD incidence among all the studied populations. Due to different living environments and genetic backgrounds, subgroup analysis based on race was also conducted, which indicated that *PON1* -108C>T polymorphism showed obvious relation with CHD susceptibility among the Asian and Caucasian populations. After that, subgroup analysis based on sample size showed that the sample size had no impact on the results. Furthermore, we also conducted sensitivity analysis, which revealed that this polymorphism was markedly correlated with CHD susceptibility. There was no obvious evidence regarding the bias of publication detected. Due to the limited sample size, our results must be further investigated.

This study did not illustrate the related mechanism of such relation. Previous study and our bioinformatics analysis found that -108T allele decreased PON1 level (8). PON1 is believed to promote the anti-inflammatory and anti-oxidant functions of lipoproteins (11). Ultimately, decreased PON1 led to the occurrence and progression of CHD. Moreover, Bouman et al. demonstrated that PON1 is the rate-limiting enzyme in the second step of clopidogrel bioactivation, in which 2-oxo-clopidogrel is hydrolyzed into the active thiol molecule. An analysis of 112 individuals revealed that the PON1 -108C>T polymorphism is the major factor affecting clopidogrel bioactivation (14).

Certain limitations must be noted in this meta-analysis. Firstly, this meta-analysis was carried out according to the non-adjusted ORs, since ORs were not available in each study and the available ORs were unadjusted relative to identical possible confounding factors, like exposure, age and sex. The insufficient information for data analysis possibly induced severe confounding bias. Secondly, the insufficient raw materials prevented us from better evaluating the possible associations between genes and the environment or between genes. Thirdly, only few studies and participants were involved in certain subgroups-based meta-analysis.

## 5 Conclusions

To sum up, this meta-analysis indicates the relationship of *PON1* -108C>T polymorphism with CHD susceptibility. More articles that estimate the impacts of interactions between genes and between genes and the environment are needed to further and comprehensively understand the correlation between *PON1* -108C>T polymorphism and CHD susceptibility.

## Data Availability

The original contributions presented in the study are included in the article/Supplementary Material, further inquiries can be directed to the corresponding author.
